# Availability of cancer survivorship support services across the National Cancer Institute Community Oncology Research Program network

**DOI:** 10.1093/jncics/pkae005

**Published:** 2024-01-24

**Authors:** Jamie M Faro, Emily V Dressler, Carol Kittel, Dori M Beeler, Shirley M Bluethmann, Stephanie J Sohl, Andrew M McDonald, Kathryn E Weaver, Chandylen Nightingale, Landscape Committee, Heather B Neuman, Chandylen L Nightingale, Susan K Parsons, Samilia Obeng-Gyasi, Mary E Cooley, Kah Poh Loh, Scott D Ramsey, Andrew M McDonald, Melyssa Foust, Christa Braun-Inglis, Wade T Kyono, Charles W Drescher, Eden G Wood, Emily V Dressler

**Affiliations:** Department of Population and Quantitative Health Science, University of Massachusetts Chan Medical School, Worcester, MA, USA; Department of Biostatistics and Data Science, Wake Forest University School of Medicine, Winston-Salem, NC, USA; Department of Biostatistics and Data Science, Wake Forest University School of Medicine, Winston-Salem, NC, USA; Department of Supportive Oncology, Levine Cancer Institute, Atrium Health, Charlotte, NC, USA; Department of Social Sciences and Health Policy, Wake Forest University School of Medicine, Winston-Salem, NC, USA; Department of Social Sciences and Health Policy, Wake Forest University School of Medicine, Winston-Salem, NC, USA; Department of Radiation Oncology, The University of Alabama at Birmingham, Heersink School of Medicine, Birmingham, AL, USA; Department of Social Sciences and Health Policy, Wake Forest University School of Medicine, Winston-Salem, NC, USA; Department of Social Sciences and Health Policy, Wake Forest University School of Medicine, Winston-Salem, NC, USA

## Abstract

**Background:**

National cancer organizations recommend provision of nutrition, physical activity, and mental health supportive services to cancer survivors. However, the availability of these services across diverse community oncology settings remains unclear.

**Methods:**

The National Cancer Institute Community Oncology Research Program (NCORP) is a national network of community oncology practices engaged in cancer research. The 2022 NCORP Landscape Assessment (5UG1CA189824) assessed individual practices’ establishment of survivorship clinics and nutrition, physical activity, and mental health services, resources, and/or referrals. Descriptive statistics summarized and logistic regression quantified the association between services, practice, and patient characteristics.

**Results:**

Of 46 NCORP community sites, 45 (98%) responded to the survey, representing 259 adult practice groups. A total of 41% had a survivorship clinic; 96% offered mental health, 94% nutrition, and 53% physical activity services, resources, and/or referrals. All 3 services were offered in various formats (eg, in-house, referrals, education) by 51% and in-house only by 25% of practices. Practices with advanced practice providers were more likely to have a survivorship clinic (odds ratio [OR] = 3.19, 95% confidence interval [CI] = 1.04 to 9.76). Practices with at least 30% Medicare patients (OR = 2.54, 95% CI = 1.39 to 4.66) and more oncology providers (OR = 1.02, 95% CI = 1.01 to 1.04) were more likely to have all 3 services in any format. Practices with at least 30% Medicare patients (OR = 3.41, 95% CI = 1.50 to 7.77) and a survivorship clinic (OR = 2.84, 95% CI = 1.57 to 5.14) were more likely to have all 3 services in-house.

**Conclusions:**

Larger oncology practices and those caring for more survivors on Medicare provided more supportive services, resources, and/or referrals. Smaller practices and those without survivorship clinics may need strategies to address potential gaps in supportive services.

Cancer survivors have unique needs for both mental and physical health that begin at diagnosis and persist throughout life (1). To further improve survivors’ long-term health, several organizations have recommended guidelines to improve survivorship care during and after treatment. The 2020 Commission on Cancer Survivorship Standard 4.8 was formed by the American Cancer Society, American Society of Clinical Oncology, National Cancer Institute, and National Cancer Policy Forum (2). The Standard recommends cancer survivorship programs identify a list of services and programs, either in-house or through referrals, and document a minimum of 3 services offered per year (3). The list of services includes, but is not limited to, physical activity programs, nutritional services, and psychological and psychiatric services. The National Comprehensive Cancer Network guidelines for healthy living also support these survivorship recommendations and reflect provision of nutrition, physical activity, and mental health services to survivors across the continuum of care, including those receiving prolonged therapy, those with chronic cancers (eg, metastatic disease), and long-term survivors (4). These recommendations are aligned with survivors’ self-reported unmet needs during and following cancer treatment, which include physical health and activity, nutrition, daily living, and mental health (5-9).

Despite these recommended guidelines, evidence shows survivors’ needs are still not adequately addressed from diagnosis through posttreatment to mitigate the late- and long-term effects of cancer (10-12). Barriers include fragmented care coordination and lack of infrastructure for survivorship services, including lack of resources and referrals (13,14). Survivorship models of care to address patient needs are related to the type of setting (ie, location, ownership type, critical access hospital designation), staffing resources, and survivor populations served (15). As the evidence for survivorship care services continues to mount, National Cancer Institute (NCI)–designated Comprehensive Cancer Centers have shown an increase in the number of support services offered, including establishment of long-term survivorship clinics (16,17). Survivorship clinics may help manage the physical and emotional changes survivors experience after cancer treatment. Attending survivorship clinics has shown improved pre- and postmental health outcomes for survivors who have completed active treatment (18,19). Community cancer programs, found in large and small community hospitals, often do not have survivorship clinics and in-house specialists, thus relying on relationships with community organizations to provide referrals (20). Survivors in rural locations have reduced access to survivorship services and specialists, and few clinical trial options, while often facing poorer health outcomes than those in urban locations (21).

The aim of this research was to assess the prevalence of existing survivorship supportive care services and format for delivering these services within practice groups participating in the NCI Community Oncology Research Program (NCORP). The NCORP network designs and conducts clinical trials in community oncology practices focused on cancer prevention, screening, surveillance, supportive care, symptom management, and care delivery. Currently, there are approximately 1000 discrete clinic locations in the United States and Puerto Rico. We examined practice-level establishment of a general survivorship clinic and availability of mental health, nutrition and physical activity support services, resources, and/or referrals. We based the assessment of these specific services on the above-described national guideline recommendations and types of services survivors have requested. We also examined relevant site characteristics associated with these outcomes.

## Methods

### Design

We examined data from the 2022 NCORP Landscape Assessment, a data collection effort coordinated through the Wake Forest NCORP Research Base. Details of the survey have been previously reported (22), and clinics had experience completing similar surveys in 2017 and 2020. In brief, NCORP supports the recruitment of patients to clinical trials from a national network of community oncology clinics (https://ncorp.cancer.gov/). NCORP is comprised of 7 research bases and 46 community sites, 14 of which are designated as minority and/or underserved community sites, having a patient population of at least 30% racial and ethnic minorities or rural residents. The NCORP Landscape Assessment solicits information on community site infrastructure, resources, and capacity for conducting research among NCORP affiliates and/or subaffiliates. The term *affiliates and/or subaffiliates* refers to the specific community oncology practice group, which could be a single clinic or a cluster of clinics that share common administration and research services. The Landscape Assessment was completed by administrators and research staff at NCORP clinics using a survey delivered through an electronic data capture tool, REDCap (23,24). This study was reviewed and deemed exempt by the institutional review board at Wake Forest University School of Medicine in Winston-Salem, North Carolina.

### Measures

Landscape Assessment questions used for the current analysis can be found in Supplementary Figure 1 (available online). Practice groups were asked if they had a general survivorship clinic available and to estimate the number of providers practicing in the general survivorship clinic. They were also asked if mental health services were available to their oncology patients. Lastly, they were asked if nutrition counseling and exercise, physical activity, and/or fitness counseling and interventions (excluding physical therapy services and research interventions) were available and, if yes, the format for availability of services (eg, in-house, referrals, education materials). The full questions and response choices for these outcomes are shown in Supplementary Figure 1 (available online).

We examined practice and patient-level characteristics, including the number of new cancer cases per year, practice ownership type, number of oncology providers and advanced practice providers, and critical access hospital designation (located in a rural or underserved area with <25 inpatient beds) (25). We excluded practices serving predominantly pediatric patients, as pediatric and adult patients’ unique needs and support services may differ. Like prior studies, practice group geographic areas were clustered into the 4 census regions including West, Midwest, Northeast, and South for analyses (26-28). We also examined patient-level characteristics for practices, such as the proportion of patients on Medicare or Medicaid, and the proportion of racial and ethnic minority patients.

### Statistical analyses

We created a composite variable identifying practices that offered all 3 services in any format, including 1) mental health services available for oncology patients or referral relationships with mental health providers in the community; 2) nutrition counseling and intervention for oncology patients; and 3) exercise, physical activity, or fitness counseling and interventions (excluding physical therapy services and research interventions) for oncology patients undergoing cancer therapies. We also created a composite variable to identify practices that offered all 3 services in-house to their patients, including 1) in-house mental health services available for oncology patients, 2) in-house nutritionist with no specialty oncology training or an in-house nutritionist with specialty oncology training, and 3) in-house exercise, wellness, and/or fitness center or an in-house cancer exercise, wellness, and/or cancer rehabilitation program.

Frequency statistics summarized practice-group characteristics and prevalence of the following primary outcomes: having a general survivorship clinic; offering mental health, nutrition, and physical activity support services; offering all 3 services in any format; and offering all 3 services in-house. Logistic regression models were constructed to examine associations with practice- and patient-level characteristics (outlined above) for having a general survivorship clinic, offering 3 services in any format, and offering 3 services in-house using stepwise model selection with entry criteria of an alpha of 0.15 with adjusted odds ratios (ORs) and 95% confidence intervals (CIs). All analyses were conducted in SAS (v.9.4, Cary, NC, USA) with a 2-sided alpha of 0.05 as the criteria for statistical significance.

## Results

### Practice and patient characteristics

The 2022 Landscape Assessment had 45 of 46 (98%) NCORP Community Sites respond to the survey, representing 517 discrete clinical locations (52%; 517 of approximately 1000 total NCORP locations) and corresponding to 271 practice groups. After excluding practice groups serving only pediatric patients, we included 259 practice groups in the analyses. Of those 259, a total of 189 (73%) are single locations, and 70 (27%) represent more than 1 location. Table 1 presents practice group characteristics. Most practices were in the Midwest region (n = 113, 44%) and owned by a large regional and/or multistate health system (n = 157, 61%), and 15% (n = 38) were identified as a critical access hospital. Of the practices, 75% (n = 194) reported that more than 30% of their patients had Medicare, and 31% (n = 78) reported that more than 30% of patients were racial and ethnic minority patients.

**Table 1.** National Institute Community Oncology Research Program oncology practice group characteristics, excluding those that serve exclusively pediatric patients (n = 259)

Practice characteristics	No. (%) or Median (IQR)
Practice group region, No. (%)	
** **Midwest	113 (44)
** **West	47 (18)
** **Northeast	25 (10)
** **South	74 (29)
Practice ownership type, No. (%)	
** **Owned by large regional and/or multistate health system	157 (61)
** **Independently owned	72 (28)
** **Other (health maintenance organization and/or payer, publicly or university owned)	30 (12)
No. of adult oncology providers,^a^^,^^b^ median (interquartile range)	11 (7-20)
** **Medical oncologists	5 (3-10)
** **Surgical oncologists	5 (3-6)
** **Radiation oncologists	2 (2-4)
** **Gynecology oncologists	2 (1-3)
Utilizing advanced practice providers, No. (%)	238 (92)
Critical access hospital,^c^ No. (%)	38 (15)
Participates in Medicare/Medicaid Oncology Care Model, No. (%)	72 (28)
≥30% new cancer patients on Medicare, No. (%)	194 (75)
≥30% new cancer patients on Medicaid, No. (%)	26 (10)
≥30% new cancer patients uninsured, No. (%)	11 (4)
≥30% of new cancer patients are racial and ethnic minority, No. (%)	78 (31)

a Sum of medical, surgical, radiation, and gynecology oncology providers.

b Fewer than 10 practice groups with missing data.

c A cluster was considered critical access if at least 1 practice identified as a critical access hospital within the larger cluster.

### Support services available

**Figure 1.**
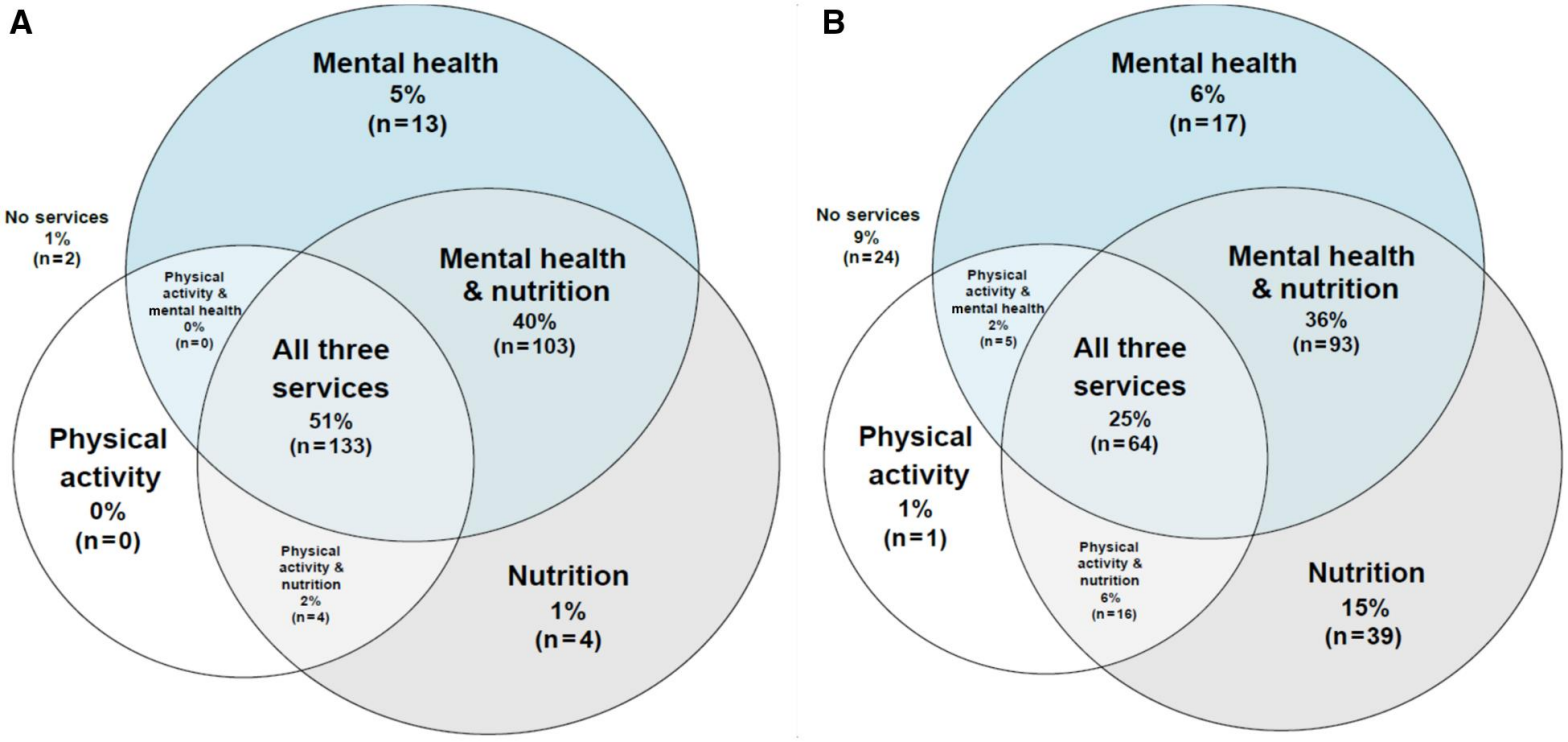
Proportion of practices with services available in any format **(A)** and in-house only **(B)**. Venn diagram showing the proportions of practices with 1, 2, or 3 services available. The sum of all numbers in each circle represents the proportion of having either mental health, physical activity, or nutrition services available. The sum of 2 numbers in each overlapping area by 2 circles represents the proportion of having 2 services available. The figure in the overlapping area in the middle represents the proportion of having all 3 services available. The proportion of practices with no support services available is located outside the circles.

Table 2 presents descriptives for type and format of each support service available. Of the practices, 41% (n = 106) had a general survivorship clinic. Most practices (69%, n = 179) offered mental health services in-house, and 27% (n = 70) referred to outside community providers. Most practices (94%, n = 244) offered nutrition counseling, with 82% (n = 212) offering in-house nutrition counseling and 55% (n = 142) offering a nutritionist with specialty oncology training. Of the practices, 53% offered physical activity counseling and/or programs, 33% (n = 86) of practices offered in-house fitness counseling and/or programs, and 24% (n = 63) offered an actual exercise and/or rehabilitation program. For composite variables, 51% (n = 133) of practices had all 3 support services available (ie, mental health, nutrition, and physical activity) in any format, while only 25% (n = 64) had all 3 support services available in-house. Figure 1 illustrates the proportion of practices offering each service independently and in combination with other services.

**Table 2.** Description of support services available in National Institute Community Oncology Research Program oncology practice groups

Service available	No. (%) (n = 259)
General survivorship clinic	106 (41)
Mental health	
** **Mental health counseling, any	249 (96)
** **Services provided in-house^a^	179 (69)
** **Referrals to community providers^a^	70 (27)
Nutrition	
** **Nutrition counseling, any	244 (94)
** **Educational materials provided by staff^a^	163 (63)
** **Telenutrition^a^	102 (39)
** **Educational materials on website^a^	56 (22)
** **Outside referral to nutritionist^a^	29 (11)
** **Other^a^^,^^b^	16 (6)
** **Nutrition counseling, in-house	212 (82)
** **In-house nutritionist with specialtyoncology training^a^	142 (55)
** **In-house nutritionist, no specialty oncologytraining^a^	92 (36)
Physical activity	
** **Fitness counseling and/or exercise program, any	137 (53)
** **Educational materials provided by staff^a^	98 (38)
** **Outside referral to a cancer exercise, wellness and/or rehabilitation program^c^	53 (20)
** **Educational materials on website^a^	34 (13)
** **Connected to an outside exercise and/or wellnessprogram^a^	31 (12)
** **Other^a^^,^^c^	7 (3)
** **Fitness counseling and/or exercise program provided in-house	86 (33)
** **In-house cancer exercise, wellness or cancerrehabilitation program^a^	63 (24)
** **In-house exercise, wellness and/or fitness center^a^	43 (17)
** **In-house tele-exercise^a^	15 (6)
Composite variables	
** **All 3 services available in any format	133 (51)
** **All 3 services available in-house	64 (25)

a Proportion of practice groups from the parent category and not from the overall sample.

b Included community programs, social media recipe sharing, integrative health, webinars, support groups, and virtual cooking demonstrations.

c Included YouTube, Zoom classes, integrative medicine, physical therapy, yoga videos, and telehealth services.

### Associations between practice and patient characteristics with support services

In the univariate models, utilizing advanced practice providers was associated with having a general survivorship clinic (OR = 3.19, 95% CI = 1.04 to 9.76). Practices with at least 30% of cancer patients covered by Medicare (OR = 2.19, 95% CI = 1.23 to 3.91) and number of oncology providers (OR = 1.02, 95% CI = 1.00 to 1.03) were associated with availability of all 3 services in any format. Practices with at least 30% of cancer patients covered by Medicare (OR = 2.89, 95% CI = 1.30 to 6.45) and having a general survivorship clinic (OR = 2.52, 95% CI = 1.41 to 4.45) were associated with availability of all 3 services in-house. We found similar results in the adjusted logistic models (Table 3). Practices that had advanced practice providers were almost 3 times as likely to report having a general survivorship clinic. Practices that had at least 30% of patients with Medicare and a greater number of oncology providers were more likely to have the 3 support services available in any format. Practices that had the 3 services available in-house were associated with at least 30% of patients with Medicare and with having a general survivorship clinic.

**Table 3.** Logistic regression model associations with having a general survivorship clinic, availability of 3 services in any format, and availability of 3 services in-house

Survivorship services	Odds ratio (95% CI)	*P*
Having a general survivorship clinic		
Utilize advanced practice providers^a^	3.19 (1.04 to 9.76)	.042
Availability of 3 services in any format		
Proportion with Medicare ≥30%^a^	2.54 (1.39 to 4.66)	.003
Number of adult oncology providers	1.02 (1.01 to 1.04)	.012
Availability of 3 services in-house		
Proportion with Medicare ≥30%^a^	3.41 (1.50 to 7.77)	.004
Have general survivorship clinic^a^	2.84 (1.57 to 5.14)	<.001

a Reference category = no. Only variables retained in the model are presented. Covariates considered included all those site and patient characteristics presented in Table 1. CI = confidence interval.

## Discussion

We examined availability of general survivorship clinics; if and how mental health, nutrition, and physical activity support services, resources, and/or referrals were available for patients at practices; and practice- and patient-level characteristics associated with these among adult NCORP practice groups. Less than half of practice groups had a general survivorship clinic. Most sites offered mental health and nutrition services, resources, and/or referrals, while just over half offered physical activity services, resources, and/or referrals. Half of the practices offered mental health, nutrition, and physical activity services, resources, and/or referrals in any format, while only one-quarter offered all 3 supportive services in-house. Practices with advanced practice providers were more likely to have general survivorship clinics than those without advanced practice providers. Practices with more than 30% of patients on Medicare and a greater number of oncology providers were more likely to offer all 3 services, resources, and/or referrals in any format, whereas practices with at least 30% of patients on Medicare and an established general survivorship clinic were more likely to offer all 3 services in-house.

We identified 106 practice groups with a general survivorship clinic. General survivorship clinics are more commonly found at higher resources settings, such as NCI-designated Comprehensive Cancer Centers (29,30). However, most of the practice groups within NCORP are not NCI-designated Cancer Centers, thus our finding of 41% offering survivorship clinics suggests the presence of these clinics may be increasing outside of higher-resourced settings. We found that practices using advanced practice providers were associated with having a general survivorship clinic; however, it is unknown what types of providers staffed this clinic. Advanced practice providers are involved in patients’ care across the cancer continuum, including survivorship care in accordance with the Institute of Medicine Standards (31,32). Survivorship care visits are billable by advanced practice providers, and evidence shows advanced practice providers–led survivorship clinics produce enough reimbursable income to cover their salaries (33). This may be a cost-effective strategy for delivering survivorship care, especially in the context of oncology physician shortages. Mental health services/referrals were offered by 96% of practice groups, with 69% offering in-house services and 27% offering community provider referrals. This high proportion of in-person and referral services is promising and may reflect the impact of a specific 2020 Commission on Cancer Standard for psychological distress screening and provision of at least a referral to services. The standard is further supported by other organizations including the American Society of Clinical Oncology’s recently revised mental health guidelines. These guidelines recommend using a stepped-care model to provide the most effective and least resource-intensive intervention based on symptom severity (34). This includes starting with education for all patients, followed by behavioral therapy and pharmacologic treatments. It will be important for future studies to examine the type of mental health services offered and how they align with these recently revised guidelines.

A high proportion (94%) of clinics reported offering nutrition services, resources, and/or referrals, including in-person nutrition counseling services (84%). This is notably higher than prior studies, including a review of 40 Comprehensive Cancer Centers that revealed only 75% offered nutrition counseling (35). Another review showed that 80% of patients receiving outpatient chemotherapy wanted nutritional counseling, and only 17% received it, with nutrition counseling patients largely including those at risk of malnutrition due to head and neck, gastrointestinal, and lung cancers (36). Although our findings indicate that nutrition services, resources, and/or referrals are available at practice groups, it is not clear how frequently these are offered to or received by patients. One potential barrier to accessing nutritional counseling includes reimbursement, as some insurances do not cover these services for survivors (37).

Just over half of the practices reported offering physical activity services, resources, and/or referrals, despite physical activity being associated with improved health outcomes after cancer diagnoses (38) and considered safe and efficacious for survivors (39,40). Several organizations recommend that providers offer referrals to physical activity in their survivorship care plans (41,42), yet few providers adhere to the recommendation. In this study, of those who reported offering physical activity services, resources, and/or referrals, 38% reported provision of educational materials to survivors by staff. Prior evidence reports that providers cite a lack of knowledge about available behavior change programs (43) and uncertainty of who refers the patient and performs the follow-up as barriers to providing recommendations and subsequent referrals (44-46). Survivors have reported lack of physical activity guidance, prescriptions, and referrals from their care team as barriers to activity (47).

We found no differences in offering support services, resources, and/or outside referrals in practices with higher and lower proportions of racial and ethnic minority patients. This was an unexpected finding, as racial and ethnic differences have been found in survivorship experience and in survivors’ obesity rates (48). However, the demographics of the patients who were offered services, resources, and/or referrals in any format within the practice remains unclear, warranting further investigation to ensure equitable access to supportive services. We noted that practices with at least 30% of patients with Medicare were associated with offering all 3 services, resources, and/or referrals in any and in-person formats. It is possible that Medicare coverage may provide better reimbursements for support services, resources, and/or referrals at these practices (49).

This study yields several implications for practice and future research focused on delivery of supportive services to cancer patients and survivors. First, it is imperative to partner with NCORP practices to understand site-specific contextual needs and barriers to support services, resources, and/or referrals. Multilevel interventions are also needed, as patient-level interventions alone may lack elements of sustainability, such as those due to the cost of services. It may be important to target smaller NCORP practices not offering any support services, education, or referrals, with the ability to provide lower intensity and pragmatic interventions. Compared with fully staffed in-house programs, lower intensity interventions may not be as efficacious; however, they can provide a greater reach to populations with greater barriers to access, thereby increasing population health promotion (50). For example, prior provider-targeted trials have used simple clinic-based electronic platforms to refer survivors to already existing physical activity programs based in the community (51). However, implementation strategies are needed to increase delivery of services, education, and referrals in formats that are feasible for their patient population and sustainable within practice. It will also be critical to enhance screening efforts to more effectively identify patients who may need support services. The National Comprehensive Cancer Network recommends patients be screened for distress at every visit using the Distress Thermometer (52). This scale focuses on concerns within the past week, such as depression and worry, food and eating, and physical abilities. This scale could be augmented with health promotion questions and serve as a gateway to proactively intervene with patients before they become distressing issues.

Landscape data used in the current study were collected primarily through a self-report survey, though respondents were asked to consult with other staff and/or databases, such as cancer registry data, when appropriate. As these data were self-reported by each practice group, there is ambiguity in how each practice group defined a given service. We lack data and comparisons on those practices that did not participate in this Landscape Assessment. This study focused on only a single cancer research network, though it does comprise a geographically diverse national sample of more than 1000 discrete clinical sites in the United States. It is unclear how results may generalize to oncology practices outside of NCORP. We also lack details on the services, resources, and/or referrals being offered, including the timing and frequency, how patients are identified, which patients receive them, in what form they receive them, if multiple forms are offered from one practice, patient utilization, and barriers to utilization.

Our findings suggest that oncology practice groups that utilize advanced practice providers were more likely to have general survivorship clinics than those that do not. We also found that practices comprised of at least 30% older survivors are more likely to provide mental health, nutrition, and physical activity supportive services, resources, and/or referrals; the format through which services are provided varies. Future research may engage NCORP sites to address expansion of support services for cancer survivors. Strategies to increase services may need to be tailored by practice to enhance pragmatism and sustainability of the given service.

## Supplementary Material

pkae005_Supplementary_Data

## Data Availability

The data underlying this article were provided by the NCORP Landscape Committee under license/by permission. Data will be shared on request to the corresponding author with permission of the NCORP Landscape Committee.
