# Supplementary material for: Availability of cancer survivorship support services across the National Cancer Institute Community Oncology Research Program network
Source: JNCI Cancer Spectr. 2024 Jan 24;8(1):pkae005. doi: 10.1093/jncics/pkae005 (PMC10868389; doi:10.1093/jncics/pkae005)
Supplement: pkae005_Supplementary_Data [file pkae005_supplementary_data.pdf]

Supplementary Figure 1. Landscape Assessment questions assessing main outcomes.

**General Survivorship Clinic**<br>Does your affiliate/subaffiliate have a General Survivorship clinic(s)?<br>__ Yes<br>__ No
**Mental Health Services, Resources, and Referrals**<br>Are mental health services available for oncology patients at your affiliate/subaffiliate?<br>__Yes<br>__No<br>___No, we do not offer at our affiliate/subaffiliate but we have referral relationships with mental health providers in the community
**Nutrition Services, Resources, and Referrals**<br>Does your affiliate/subaffiliate offer nutrition counseling and intervention for oncology patients?<br>__Yes<br>__No<br>a. If yes, how does nutrition counseling occur? (Select all that apply)<br>___In house nutritionist, no specialty oncology training<br>___In house nutritionist with specialty oncology training<br>___Telenutrition (i.e., nutrition services offered remotely by in house providers or staff with technology)<br>___Outside referral to nutritionist<br>___Educational materials (e.g., pamphlets) provided by our staff<br>___Educational materials on our website<br>_____Other, please specify:
**Physical Activity Services, Resources, and Referrals**<br>Does your affiliate/subaffiliate offer exercise, physical activity, or fitness counseling and interventions (excluding physical therapy services and research interventions) for oncology patients undergoing cancer therapies?<br>__Yes<br>__No<br>a. If yes, what services are offered? (Select all that apply)<br>___In house exercise/wellness or fitness center<br>___In house cancer exercise/wellness or cancer rehabilitation program<br>___In house tele-exercise<br>___Outside referral to a cancer exercise/wellness or cancer rehabilitation program<br>__Connected to an outside exercise/wellness program<br>___Educational materials provided by our staff<br>___Educational materials on our website<br>_____Other, please specify:
